# Monocyte-to-albumin ratio as a novel predictor of long-term adverse outcomes in patients after percutaneous coronary intervention

**DOI:** 10.1042/BSR20210154

**Published:** 2021-06-29

**Authors:** Zeng-Lei Zhang, Qian-Qian Guo, Jun-Nan Tang, Jian-Chao Zhang, Meng-Die Cheng, Feng-Hua Song, Zhi-Yu Liu, Kai Wang, Li-Zhu Jiang, Lei Fan, Xiao-Ting Yue, Yan Bai, Xin-Ya Dai, Ru-Jie Zheng, Ying-Ying Zheng, Jin-Ying Zhang

**Affiliations:** 1 Department of Cardiology, First Affiliated Hospital of Zhengzhou University, Zhengzhou, Henan, China; 2 Key Laboratory of Cardiac Injury and Repair of Henan Province, Zhengzhou, Henan, China

**Keywords:** coronary artery disease, monocyte-to-albumin ratio, mortality, percutaneous coronary intervention, prognosis

## Abstract

**Background:** Monocyte count and serum albumin (Alb) have been proven to be involved in the process of systemic inflammation. Therefore, we investigated the prognostic value of monocyte-to-albumin ratio (MAR) in patients who underwent percutaneous coronary intervention (PCI).

**Methods:** We enrolled a total of 3561 patients in the present study from January 2013 to December 2017. They were divided into two groups according to MAR cut-off value (MAR < 0.014, *n*=2220; MAR ≥ 0.014, *n*=1119) as evaluated by receiver operating characteristic (ROC) curve. The average follow-up time was 37.59 ± 22.24 months.

**Results:** The two groups differed significantly in the incidences of all-cause mortality (ACM; *P<*0.001), cardiac mortality (CM; *P<*0.001), major adverse cardiovascular events (MACEs; *P=*0.038), and major adverse cardiovascular and cerebrovascular events (MACCEs; *P=*0.037). Multivariate Cox regression analyses revealed MAR as an independent prognostic factor for ACM and CM. The incidence of ACM increased by 56.5% (hazard ratio [HR] = 1.565; 95% confidence interval [CI], 1.086–2.256; *P=*0.016) and that of CM increased by 76.3% (HR = 1.763; 95% CI, 1.106–2.810; *P=*0.017) in patients in the higher-MAR group. Kaplan–Meier survival analysis suggested that patients with higher MAR tended to have an increased accumulated risk of ACM (Log-rank *P<*0.001) and CM (Log-rank *P<*0.001).

**Conclusion:** The findings of the present study suggested that MAR was a novel independent predictor of long-term mortality in patients who underwent PCI.

## Introduction

Despite advances in prevention, diagnosis, and treatment of cardiovascular disease over the past two decades, it is still the leading cause of deaths in both developed and developing countries, and coronary artery disease (CAD) makes the greatest contribution to CVD [1]. CAD is the consequence of complex, disordered metabolic processes that are inflammatory in nature [2]. Accumulating evidence has revealed that inflammation plays a vital role in the pathogenesis of CAD and atherosclerosis [2–4].

Monocytes are innate immunity’s primary players and comprise 10% of human blood leukocytes [5]. They remain in a steady state in blood vessels and transmigrate across endothelium into the vascular intima when stimulated by inflammatory cytokines. Here, monocytes differentiate into macrophages that uptake lipids to form foam cells, which comprise a significant proportion of atherosclerotic plaque [6,7]. In addition, monocytes promote destabilization of the fibrous cap, leading to plaque rupture [8]. Berg et al. (2012) showed that percentage and number of classical monocytes could predict cardiovascular events [9]. Albumin (Alb) as a negative acute-phase protein, is considered a measure of the intensity of the infection-triggered inflammatory response [10], because inflammatory conditions can decrease Alb levels by altering hepatic-protein metabolism and inducing capillary leakage [11–13]. Population-based studies have shown that lower Alb is associated with development of atherosclerosis and initiation of MI [14–16]. In addition, Kurtul et al. (2016) demonstrated that decreased Alb level is an independent predictor of high syntax score and in-hospital mortality in patients with acute coronary syndrome (ACS) [17].

In particular, monocytes and Alb are associated with progression of CAD [6–8,18]. While much attention has been paid to studies on monocytes and Alb, there is still a gap in our knowledge on the relationship of each with monocyte-to-albumin ratio (MAR). Therefore, in the present study, we examined the MAR index of CAD patients who had undergone percutaneous coronary intervention (PCI), and below we discuss the ability of this index to predict long-term clinical outcomes.

## Methods

### Study design and population

In the present study, we included a total of 3561 CAD patients admitted to the Clinical Outcomes and Risk Factors of Patients with Coronary Heart Disease after PCI (CORFCHD-ZZ; identifier: ChiCTR1800019699) study. This large, single-center retrospective cohort study included 3561 CAD patients diagnosed by coronary angiography (CAG), hospitalized at the First Affiliated Hospital of Zhengzhou University (Zhengzhou, China) during 2013–2017 and having received at least one stent via implantation. Exclusion criteria included severe valvular heart disease, decompensated heart failure, pulmonary heart disease, rheumatic heart disease, serious renal or hepatic disease, pernicious anemia, infectious diseases, and malignancies. The present study complied with the Declaration of Helsinki and was approved by the Ethics Committee of the First Affiliated Hospital of Zhengzhou University.

**Figure 1:**
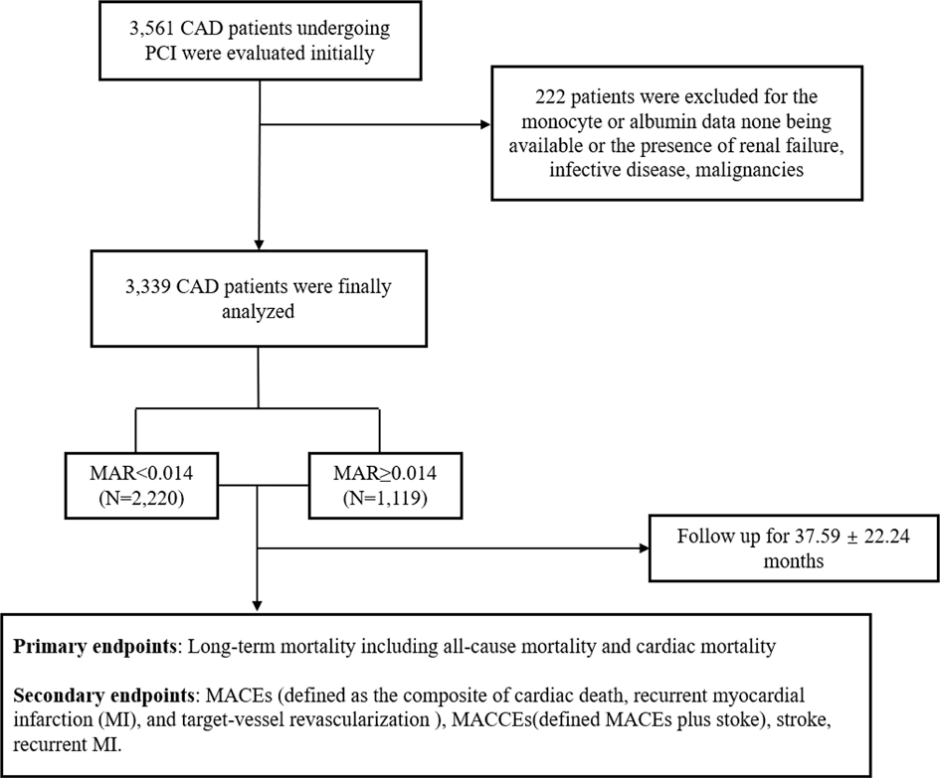
The flowchart of patients’ enrollment

Of the 3561 patients, we initially evaluated in order to analyze the correlation between MAR (higher MAR means an increase in monocytes and a decrease in Alb) and clinical outcomes in CAD patients who underwent PCI, we excluded 222 for unavailable monocyte or Alb data or the presence of renal failure, infectious diseases, or malignancies. Ultimately, we enrolled 3339 patients in the present study. Figure 1 shows the flowchart of the inclusion and exclusion criteria.

### Definitions

According to American Heart Association (AHA) recommendations [19], hypertension is defined as (i) a definite history of hypertension and active treatment with antihypertensive drugs, or (ii) systolic blood pressure ≥ 140 mm Hg and/or diastolic blood pressure ≥ 90 mm Hg on at least three resting measurements on at least two separate healthcare visits. Type 2 diabetes was defined as (i) any treatment with glucose-lowering agents, (ii) fasting plasma glucose ≥ 7.1 mmol/l, or (iii) 2-h post-load glucose ≥ 11.1 mmol/l [20]. Diagnostic criteria for hyperlipidemia were mainly obtained from the *Guidelines for Chinese Adult Dyslipidemia Prevention and Treatment* (2016) [21]. Smoking status was one of the following: current smoker, former smoker, or never-smoker. Subjects who had been regularly using tobacco in the previous 6 months were considered current smokers. Drinking status was defined as any consumption of alcohol in the previous 6 months.

### Clinical and demographic characteristic collection

Patients’ peripheral venous-blood samples were obtained on admission to the inpatient ward and subjected to tests prior to PCI. We collected data on clinical and demographic characteristics, including age, sex, history of hypertension, family history of CAD, and smoking and drinking status, from medical records. We also noted imaging and laboratory data, including creatinine (Cr), uric acid (UA), lipid parameters, and angiographic results. Laboratory measurements for biomarkers are shown in Supplementary Table S1. The use of β-blockers, angiotensin-converting enzyme inhibitors (ACEIs), angiotensin II receptor blockers (ARBs), statins, aspirin, clopidogrel, and calcium channel blockers (CCBs) was carefully recorded during the follow-up period.

### Endpoints

The primary endpoint was long-term mortality, including all-cause mortality (ACM) and cardiac mortality (CM). Secondary endpoints were major adverse cardiovascular events (MACEs), defined as the combination of cardiac death, recurrent MI, and target vessel reconstruction (TVR) [22]; and major adverse cardiac and cerebrovascular events (MACCEs), which were defined as MACEs plus stroke [23,24]. Briefly, we considered a death to be cardiac unless a definite non-cardiac cause was identified. Recurrent MI was defined as (i) one new Q wave and an increased concentration of creatine kinase isoenzyme MB (CK-MB) to more than five-times the upper limit of the normal range within 48 h after PCI; (ii) new Q waves; or (iii) an increase in CK-MB concentration exceeding the upper limit of the normal range plus ischemic symptoms or signs if occurring >48 h after PCI, as described previously [25]. TVR was defined as any repetitive revascularization in a treated vessel with (i) ≥50% stenosis in diameter accompanied by ischemic signs or symptoms or (ii) ≥ 70% stenosis in the absence of ischemic signs or symptoms [25]. Stroke was defined as a sudden onset of vertigo, numbness, aphasia, or dysarthria caused by cerebrovascular disease, including hemorrhage, embolism, thrombosis, or aneurysm rupture and persisting for >24 h [25]. All the incidents were determined by an adjudication committee, and all the events by an event adjudication committee, who were blinded to the group of patients.

### Follow-up

In our study, all of the enrolled patients received regular follow-ups, which were conducted during office visits or by telephone interview, for at least 18 months. For the duration of follow-up, an independent group of clinical physicians carefully checked and verified all events.

### Statistical analysis

We analyzed all data using SPSS version 23.0 (IBM Corp., Armonk, NY, U.S.A.). Enrolled patients were grouped into two categories based on MAR value (MAR < 0.014, MAR ≥ 0.014). Continuous data are presented as mean ± standard deviation (SD), and categorical variables are presented as frequencies and percentages. We analyzed MAR as a continuous variable categorized into two groups based on the MAR cut-off value of 0.014, which we determined by analyzing the receiver operating characteristic (ROC) curve for the baseline data of the study population. The ROC curve was determined to predict cumulative incidence of events. To compare differences in parametric continuous variables, we used Student’s *t* tests; to compare differences in nonparametric continuous variables, we used Mann–Whitney *U* tests. Chi-square (χ^2^) tests were used for comparisons of categorical variables. We used Kaplan–Meier analysis to calculate cumulative incidence rates of long-term outcomes and the log-rank test for comparisons between groups. Multivariate Cox regression analysis was performed to examine the predictive value of MAR for outcomes throughout the 5-year follow-up period. Finally, we calculated hazard ratios (HRs) and 95% confidence intervals (CIs). *P*-values were two-sided and were considered significant when *P<*0.05.

## Results

### Baseline characteristics

**Figure 2:**
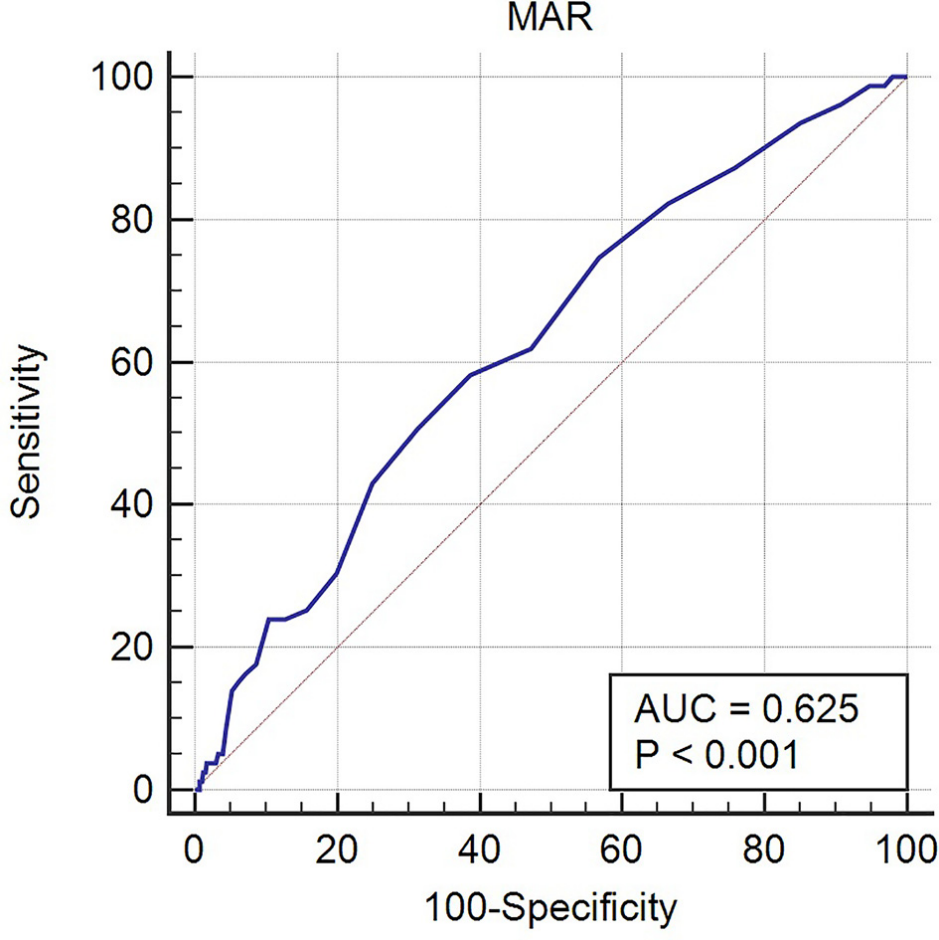
The ROC curve of MAR

In the present study, we enrolled 3339 patients who had undergone PCI. Average follow-up time was 37.59 ± 22.24 months. Sensitivity and specificity were 50.63 and 68.67%, respectively, and Youden’s index, which we calculated in accordance with a previous study [26], was 0.193. Area under the curve (AUC) was 0.625 (0.609–0.642; *P*<0.001), and the cut-off value was 0.014 (Figure 2). Participants were categorized into two groups according to MAR cut-off value: a lower-MAR group (MAR < 0.014, *n*=2220) and a higher-MAR group (MAR ≥ 0.014, *n*=1119). All of the baseline data are shown in Table 1. Monocyte counts were respectively 0.43 ± 0.10 and 0.77 ± 0.41 in these two groups, while Alb levels were respectively 41.65 ± 4.11 and 39.14 ± 4.92. Age; sex; smoking; heart rate; monocyte count; Alb level; Cr; UA; high-density lipoprotein cholesterol (HDL-C); and the use of CCB, statin, and aspirin all differed significantly between the two groups (all *P<*0.05). In ACS patients, we found that age, sex, smoking, monocyte count, Alb level, Cr, UA, HDL-C, and use of statin and aspirin were significantly different between the groups. In stable CAD patients, we found that sex, smoking, monocyte count, Alb level, Cr, UA, HDL-C, and use of CCB and statin were significantly different between the groups.

**Table 1:** Baseline characteristics

Variables	Total				ACS				Stable CAD			
	MAR < 0.014 (*n*=2220)	MAR ≥ 0.014 (*n*=1119)	χ^2^ or *t*	*P*-value	MAR < 0.014 (*n*=1698)	MAR ≥ 0.014 (*n*=849)	χ^2^ or *t*	*P*-value	MAR < 0.014 (*n*=522)	MAR ≥ 0.014 (*n*=270)	χ^2^ or *t*	*P*-value
Age, years	62.74 ± 10.47	64.37 ± 10.92	−4.189	**<0.001**	62.81 ± 10.36	64.75 ± 10.50	−4.436	**<0.001**	62.52 ± 10.80	63.18 ± 12.07	−0.758	0.449
Gender (male), *n* (%)	1441 (64.9)	858 (76.7)	48.024	**<0.001**	1105 (65.1)	651 (76.7)	35.582	**<0.001**	336 (64.4)	207 (76.7)	12.488	**<0.001**
Smoking, *n* (%)	620 (27.9)	397 (35.5)	20.023	**<0.001**	480 (28.3)	294 (34.6)	10.824	**0.001**	140 (26.8)	103 (38.1)	10.738	**0.001**
Alcohol drinking, *n* (%)	340 (15.3)	200 (17.9)	3.590	0.058	264 (15.5)	149 (17.6)	1.670	0.196	76 (14.6)	51 (18.9)	2.477	0.115
Diabetes, *n* (%)	514 (23.2)	273 (24.4)	0.639	0.424	400 (23.6)	210 (24.7)	0.431	0.511	114 (21.8)	63 (23.3)	0.229	0.632
Hypertension, *n* (%)	1242 (55.9)	614 (54.9)	0.349	0.555	946 (55.7)	478 (56.3)	0.080	0.778	296 (56.7)	136 (50.4)	2.880	0.090
Heart rate, rpm	74.10 ± 20.38	75.55 ± 11.92	−2.150	**0.032**	74.12 ± 20.59	75.68 ± 12.05	−1.863	0.063	74.10 ± 10.32	75.13 ± 11.52	−1.267	0.205
Monocytes, 10^9^/l	0.43 ± 0.10	0.77 ± 0.41	−27.04	**<0.001**	0.43 ± 0.10	0.77 ± 0.39	−33.322	**<0.001**	0.44 ± 0.09	0.78 ± 0.47	−11.829	**<0.001**
Alb, g/l	41.65 ± 4.11	39.14 ± 4.92	14.70	**<0.001**	41.64 ± 4.27	39.25 ± 4.71	12.882	**<0.001**	41.70 ± 3.55	38.82 ± 5.52	14.707	**<0.001**
Cr, μmol/l	70.39 ± 24.69	77.69 ± 51.08	−5.547	**<0.001**	70.42 ± 23.05	76.02 ± 29.67	−5.218	**<0.001**	70.29 ± 29.46	82.97 ± 89.76	−2.246	**0.025**
UA, mmol/l	294.81 ± 82.33	307.50 ± 91.96	−4.017	**<0.001**	294.83 ± 81.99	304.73 ± 93.49	−2.609	**0.006**	294.74 ± 83.49	316.27 ± 86.50	−3.371	**0.001**
TG, mmol/l	1.69 ± 1.17	1.62 ± 1.02	1.778	0.075	1.68 ± 1.16	1.59 ± 0.95	1.805	0.071	1.74 ± 1.19	1.69 ± 1.22	0.482	0.630
TC, mmol/l	3.92 ± 1.03	3.86 ± 1.01	1.608	0.108	3.93 ± 1.03	3.86 ± 1.02	1.513	0.130	3.91 ± 1.03	3.87 ± 1.01	0.582	0.559
LDL-C, mmol/l	2.40 ± 0.84	2.40 ± 0.85	0.184	0.854	2.40 ± 0.85	2.40 ± 0.85	0.053	0.958	2.39 ± 0.81	2.37 ± 0.83	0.287	0.774
HDL-C, mmol/l	1.06 ± 0.30	1.00 ± 0.27	4.621	**<0.001**	1.05 ± 0.31	1.00 ± 0.26	4.192	**<0.001**	1.06 ± 0.29	1.02 ± 0.29	1.986	**0.047**
CCB, *n* (%)	409 (18.4)	171 (15.3)	5.117	**0.024**	296 (17.4)	133 (15.7)	1.261	0.261	113 (21.6)	38 (14.1)	6.615	**0.010**
β-blocker, *n* (%)	1134 (51.1)	553 (49.4)	0.822	0.365	854 (50.3)	422 (49.7)	0.079	0.779	280 (53.6)	131 (48.5)	1.870	0.172
ACEI or ARB, *n* (%)	633 (28.5)	304 (27.2)	0.668	0.414	456 (26.9)	225 (26.5)	0.036	0.849	177 (33.9)	79 (29.3)	1.758	0.185
Statin, *n* (%)	1804 (81.3)	856 (76.5)	10.425	**0.001**	1379 (81.2)	647 (76.2)	8.717	**0.003**	425 (81.4)	209 (77.4)	1.792	0.181
Aspirin, *n* (%)	1878 (84.6)	896 (80.1)	10.827	**0.001**	1425 (83.9)	677 (79.7)	6.863	**0.009**	453 (86.8)	219 (81.1)	4.451	**0.035**
Clopidogrel, *n* (%)	529 (23.8)	281 (25.1)	0.666	0.414	405 (23.9)	219 (25.8)	1.156	0.282	124 (23.8)	62 (23.0)	0.062	0.803

Abbreviations: LDL-C, low-density lipoprotein cholesterol; TC, total cholesterol; TG, triglyceride. The boldfaced values indicate *P*<0.05.

### Clinical outcomes

**Figure 3:**
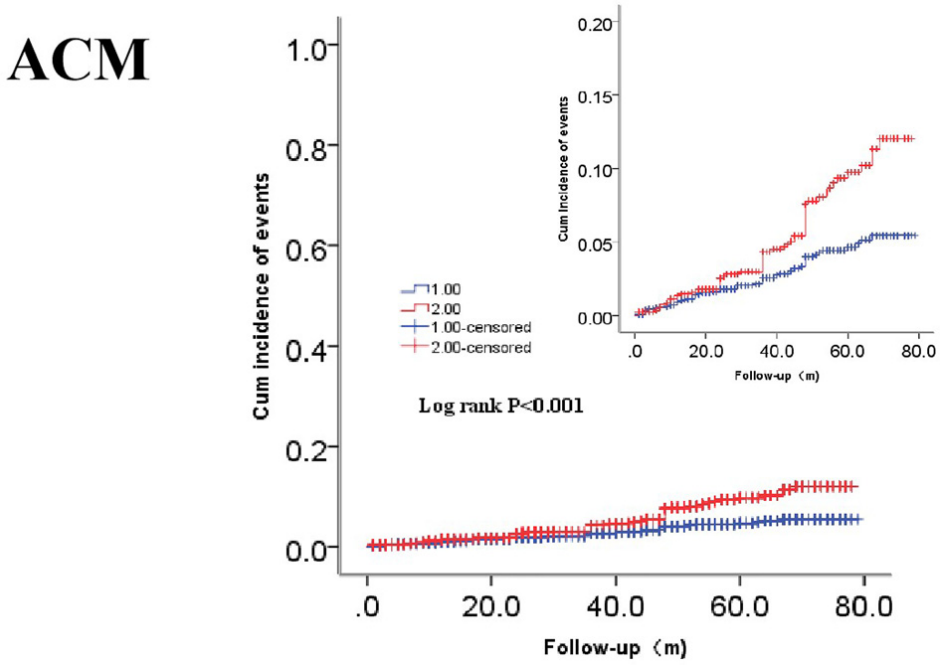
Cumulative Kaplan–Meier estimates of the time to the first adjudicated occurrence of ACM The red line indicates higher MAR and the blue line indicates lower MAR.

**Figure 4:**
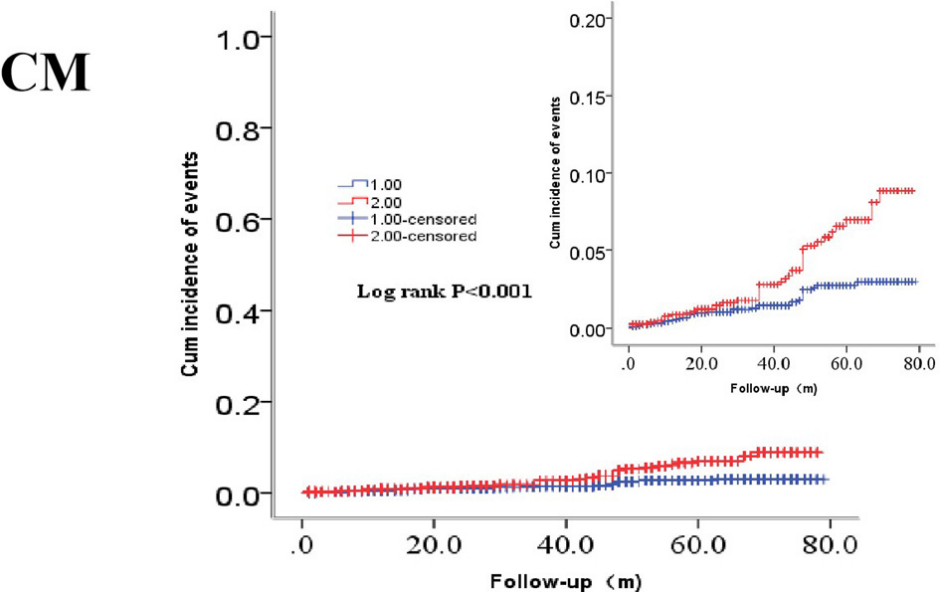
Cumulative Kaplan–Meier estimates of the time to the first adjudicated occurrence of CM The red line indicates higher MAR and the blue line indicates lower MAR.

Clinical outcomes are shown in Table 2. In terms of primary endpoints, the incidence of ACM was 2.9% in the lower-MAR group and 5.5% in the higher-MAR group (*P<*0.001), while that of CM also differed significantly between the groups (1.7 vs 3.8%, *P<*0.001). In terms of secondary endpoints, the incidences of MACEs and MACCEs showed significant differences between the groups (10.5 vs 13.0%, *P=*0.038; 13.8 vs 16.5%, *P=*0.037, respectively). However, we saw no significant between-group difference in the incidence of stroke (3.8 vs 3.8%, *P=*0.965) or recurrent MI (2.9 vs 2.6%, *P=*0.629). Kaplan–Meier survival analysis suggested that CAD patients with higher MAR tended to have an increased accumulated risk of ACM and CM (Log-rank *P<*0.001 and *P<*0.001, respectively; Figures 3 and 4).

**Table 2:** Outcomes comparison between two groups

Variables	Total				ACS				Stable CAD			
	MAR < 0.014 (*n*=2220)	MAR ≥ 0.014 (*n*=1119)	*χ*^2^	*P*-value	MAR < 0.014 (*n*=1698)	MAR ≥ 0.014 (*n*=849)	*χ*^2^	*P*-value	MAR < 0.014 (*n*=522)	MAR ≥ 0.014 (*n*=270)	*χ*^2^	*P*-value
**Primary endpoints**												
ACM, *n* (%)	65 (2.9)	62 (5.5)	13.881	**<0.001**	48 (2.8)	47 (5.5)	11.568	**0.001**	17 (3.3)	15 (5.6)	2.426	0.119
CM, *n* (%)	37 (1.7)	42 (3.8)	14.024	**<0.001**	28 (1.6)	31 (3.7)	10.029	**0.002**	9 (1.7)	11 (4.1)	3.992	**0.046**
**Secondary endpoints**												
MACEs, *n* (%)	234 (10.5)	145 (13.0)	4.321	**0.038**	172 (10.1)	111 (13.1)	4.969	**0.026**	62 (11.9)	34 (12.6)	0.085	0.770
MACCEs, *n* (%)	307 (13.8)	185 (16.5)	4.329	**0.037**	228 (13.4)	139 (16.4)	3.979	**0.046**	79 (15.1)	46 (17.0)	0.485	0.486

The boldfaced values indicate *P*<0.05.

Subgroup analysis suggested a significant difference in the incidences of ACM (2.8 vs 5.5%, *P=*0.001), CM (1.6 vs 3.7%, *P=*0.002), MACEs (10.1 vs 13.1%, *P=*0.026), and MACCEs (13.4 vs 16.4%, *P=*0.046) between the lower-MAR group and the higher-MAR group for ACS patients. For stable CAD patients, we found significant differences in the incidence of CM (1.7 vs 4.1%, *P=*0.046) between the lower-MAR and higher-MAR groups.

We created a univariate model for each of the predictor variables and entered the variables that were significant (*P<*0.05) in the univariate Cox models into multivariate Cox regression analysis. In multivariate Cox regression analysis, after we had adjusted for traditional clinical prognostic factors including age, gender, smoking, heart rate, total cholesterol (TC), low-density lipoprotein cholesterol (LDL-C), HDL-C, UA, history of diabetes, and hypertension that were significant in the univariate Cox models, we found that MAR index could predict poor clinical outcomes. During long-term follow-up compared with the lower-MAR group, the risk of ACM and CM increased by 56.5% (HR = 1.565; 95% CI, 1.086–2.256; *P=*0.016) and 76.3% (HR = 1.763; 95% CI, 1.106–2.810; *P=*0.017), respectively, in the higher-MAR group (Tables 3 and 4). However, after adjusting for the above-mentioned factors in multivariate Cox regression analysis, we did not find significant differences in the risk of MACEs (HR = 1.164; 95% CI, 0.935–1.449; *P=*0.174) or MACCEs (HR = 1.162; 95% CI, 0.959–1.408; *P=*0.126) between the two groups (Tables 5 and 6).

**Table 3:** Cox regression analysis results for ACM

Variables	B	SE	Wald	*P*	HR (95% CI)
Age (years)	0.070	0.010	51.325	<0.001	1.073 (1.052–1.094)
Gender (male)	−0.363	0.243	2.243	0.134	0.695 (0.432–1.119)
Smoking	0.045	0.215	0.043	0.835	1.046 (0.687–1.592)
Heart rate	0.002	0.002	0.679	0.410	1.002 (0.997–1.006)
TC	0.283	0.126	5.090	0.024	1.327 (1.038–1.698)
LDL-C	−0.294	0.162	3.301	0.069	0.745 (0.542–1.023)
HDL-C	−0.365	0.367	0.989	0.320	0.695 (0.339–1.425)
UA	0.002	0.001	2.220	0.136	1.002 (1.000–1.004)
Diabetes	0.473	0.200	5.621	0.018	1.605 (1.085–2.374)
Hypertension	0.108	0.194	0.309	0.578	1.114 (0.761–1.629)
MAR	0.448	0.187	5.766	0.016	1.565 (1.086–2.256)

**Table 4:** Cox regression analysis results for CM

Variables	B	SE	Wald	*P*	HR (95% CI)
Age (years)	0.065	0.012	28.764	<0.001	1.067 (1.042–1.093)
Gender (male)	−0.714	0.344	4.309	0.038	0.490 (0.249–0.961)
Smoking	0.128	0.258	0.246	0.620	1.137 (0.685–1.886)
Heart rate	0.001	0.003	0.110	0.740	1.001 (0.995–1.007)
TC	0.150	0.196	0.588	0.443	1.162 (0.792–1.704)
LDL-C	−0.139	0.237	0.341	0.559	0.871 (0.547–1.387)
HDL-C	−0.555	0.491	1.277	0.258	0.574 (0.219–1.503)
UA	0.003	0.001	4.790	0.029	1.003 (1.000–1.005)
Diabetes	0.542	0.254	4.569	0.033	1.730 (1.046–2.827)
Hypertension	0.080	0.247	0.106	0.744	1.084 (0.668–1.757)
MAR	0.567	0.238	5.680	0.017	1.763 (1.106–2.810)

**Table 5:** Cox regression analysis results for MACEs

Variables	B	SE	Wald	*P*	HR (95% CI)
Age (years)	0.007	0.005	1.898	0.168	1.007 (0.997−1.018)
Gender (male)	−0.090	0.142	0.402	0.526	0.914 (0.693−1.206)
Smoking	−0.009	0.126	0.005	0.941	0.991 (0.773−1.269)
Heart rate	0.001	0.002	0.076	0.783	1.001 (0.996−1.005)
TC	−0.022	0.114	0.039	0.844	0.978 (0.782−1.222)
LDL-C	0.140	0.131	1.150	0.284	1.151 (0.890−1.487)
HDL-C	−0.294	0.223	1.737	0.188	0.745 (0.481−1.154)
UA	<0.001	0.001	0.503	0.478	1.000 (0.999−1.002)
Diabetes	0.088	0.127	0.477	0.490	1.092 (0.851−1.400)
Hypertension	0.011	0.110	0.010	0.920	1.011 (0.814−1.255)
MAR	0.152	0.112	1.849	0.174	1.164 (0.935−1.449)

**Table 6:** Cox regression analysis results for MACCEs

Variables	B	SE	Wald	*P*	HR (95% CI)
Age (years)	0.010	0.005	4.591	0.032	1.010 (1.001−1.019)
Gender (male)	–0.056	0.122	0.210	0.647	0.946 (0.745−1.201)
Smoking	–0.075	0.113	0.439	0.508	0.928 (0.743−1.158)
Heart rate	0.002	0.001	3.804	0.051	1.002 (1.000−1.004)
TC	–0.175	0.115	2.312	0.128	0.839 (0.670−1.052)
LDL-C	0.292	0.129	5.084	0.024	1.339 (1.039−1.726)
HDL-C	–0.110	0.193	0.325	0.569	0.896 (0.613−1.308)
UA	<0.001	0.001	0.592	0.442	1.000 (0.999−1.002)
Diabetes	0.114	0.110	1.066	0.302	1.120 (0.903−1.390)
Hypertension	0.118	0.098	1.473	0.225	1.126 (0.930−1.363)
MAR	0.150	0.098	2.338	0.126	1.162 (0.959−1.408)

## Discussion

The present study investigated the prognostic value of MAR in CAD patients who had undergone PCI and found that a higher MAR was independently associated with adverse outcomes. High MAR increased the risk of long-term ACM by 56.5% and that of CM by 76.3%. To reduce the risk of confounding factors, we adjusted a comprehensive list of characteristics that influence the risk of cardiovascular events to examine the association between MAR and clinical outcomes. To the best of our knowledge, the present study is the first to investigate the relationship between the MAR index and adverse outcomes in CAD patients after PCI.

In recent years, many novel biomarkers such as genetic variation [27], micro-ribonucleic acids [28], and long noncoding RNAs [29] have been found useful in evaluating and diagnosing early damage to myocardium in clinical practice. However, limited ability to predict outcomes, as well as complicated and expensive detection methods, make these tools unavailable for clinical practice. More recently, the prognostic role of hematological biomarkers such as γ-glutamyl transpeptidase-to-platelet ratio [30], hemoglobin [31], triglyceride (TG)/HDL-C ratio [32], and red blood cell distribution width [33] in patients with CVD has been recognized, as they have been demonstrated to be efficient in predicting outcomes in CAD patients. Overwhelming evidence supports the view that monocytes and monocyte-derived macrophages, as basic cell components of the innate immune system, play pivotal roles in coronary-plaque progression [6–8]. Studies have demonstrated that monocytes are associated with an increased risk of cardiovascular events [34]. Similarly, Berg et al. (2012) [9] showed that the percentage and number of monocytes could predict cardiovascular events. Moreover, monocyte count has been reported to be an independent predictor of common carotid atherosclerosis in healthy subjects [35,36]. Decreased Alb level, associated with the chronic nature of the disease, might represent inflammatory status instead of nutritional condition [10]. Kurtul et al. (2016) reported that decreased Alb level is associated with increased incidence of in-hospital mortality in patients with ACS [17].

In the present study, we observed increased accumulated risks of ACM and CM in the higher-MAR group, which had higher monocyte counts and lower Alb concentrations than the lower-MAR group. Some mechanisms could explain the association between increased MAR and adverse clinical events in these patients. Atherosclerosis is considered a chronic, lasting inflammatory process of the arteries that is like other inflammatory diseases characterized by proliferation and infiltration of immune cells, including monocytes, macrophages, and neutrophils [2–3]. Circulating monocytes are recruited into the arterial wall and subintima, differentiate into macrophages, result in foam cells via lipid uptake, and initiate the atherosclerotic process. They then activate the production of pro-inflammatory cytokines (e.g., tumor necrosis factor α, interleukins-1 and -6, matrix metalloproteinases, and reactive oxidative species), all of which play major roles in the initiation, formation, and rupture of atherosclerotic plaque [37,38]. In contrast, decreased Alb level, which is influenced by factors such as synthesis, clearance and dilution [39], is associated with increased risks of morbidity and mortality in a range of CVDs [40–42]. Under inflammatory conditions, Alb concentration is affected by decreased hepatic synthesis, increased leakage into the interstitial space, and catabolism. Therefore, the decreased Alb levels in the higher MAR group might indicate the intensity of the inflammatory response, which has been demonstrated to have a close relationship with adverse cardiovascular events [43].

Of note, while several studies have confirmed the potential of monocytes and Alb in predicting long-term outcomes of CVDs, no attention has been paid to MAR, leaving a major gap in our knowledge. Fortunately, our study showed that MAR is a novel, powerful, available, and effective predictor of CAD outcomes after PCI, and it could be a perfect supplement to hematological biomarkers in predicting adverse outcomes in such patients. Furthermore, because MAR consists of two inflammation-related variables, monocyte count and Alb concentration, it could lead to more reliable results.

There were some limitations to our study. First, we did not monitor dynamic changes in these variables, which is unable to assess the association between their ranges of variation and outcomes of CAD patients after PCI. Second, our study did not collect data on infarct size, number and degree of stenotic vessels, or complete versus partial vascularization, nor did it evaluate the influence of these factors on prognosis. This makes it difficult to use our results to evaluate the power of MAR to predict outcomes of patients with different severities of CAD. Third, we failed to collect data on inflammatory markers such as Cluster of Differentiation 4-positive (CD4^+^) T cells that contribute to inflammatory response in CAD. In addition, the ratio is truly influenced by the two variables individually, which may exaggerate the predictive value of MAR. To fully demonstrate the association between baseline MAR and outcomes of CAD after PCI, our study needs further verification by a prospective multicenter study. We expect to see more research on the mechanism of MAR in the future.

## Conclusion

In conclusion, our findings suggested that the MAR was a sensitive, reliable, and effective predictor of adverse outcomes in CAD patients after PCI.

## Trial Registration

Chinese Clinical Trial Registry. ChiCTR-ORC-16010153. Registration date: 24 November 2018; http://www.chictr.org.cn/index.aspx

## Supplementary Material

Supplementary Table S1

## Data Availability

The data will not be shared, because the identified participants information is included in the data.

## Abbreviations

ACM: all-cause mortality
ACS: acute coronary syndrome
Alb: albumin
CAD: coronary artery disease
CCB: calcium channel blocker
CI: confidence interval
CM: cardiac mortality
Cr: creatinine
HDL-C: high-density lipoprotein cholesterol
HR: hazard ratio
LDL-C: low-density lipoprotein cholesterol
MACCE: major adverse cardiovascular and cerebrovascular event
MACE: major adverse cardiovascular event
MAR: monocyte-to-albumin ratio
PCI: percutaneous coronary intervention
ROC: receiver operating characteristic
TVR: target vessel reconstruction
UA: uric acid
