# Supplementary material for: Monocyte-to-albumin ratio as a novel predictor of long-term adverse outcomes in patients after percutaneous coronary intervention
Source: Biosci Rep. 2021 Jun 29;41(7):BSR20210154. doi: 10.1042/BSR20210154 (PMC8243340; doi:10.1042/BSR20210154)
Supplement: Supplementary Table S1 [file BSR-2021-0154_supp.pdf]

Supplementary table 1 Laboratory measurements for biomarkers

Variables	laboratory measurements
Monocytes, 10^9/L	0.1-0.6
Albumin, g/L	35-50
Cr, umol/L	20-115
UA, mmol/L	140-360
TG, mmol/L	<1.7
TC, mmol/L	<5.2
LDL-C, mmol/L	<3.61
HDL-C, mmol/L	>0.91
